# Positive and negative effects of mesograzers on early‐colonizing species in an intertidal rocky‐shore community

**DOI:** 10.1002/ece3.2323

**Published:** 2016-07-22

**Authors:** Daniela Tejada‐Martinez, Daniela N. López, César C. Bonta, Roger D. Sepúlveda, Nelson Valdivia

**Affiliations:** ^1^ Doctorado en Ciencias, mención en Ecología y EvoluciónFacultad de CienciasUniversidad Austral de ChileCampus Isla TejaValdiviaChile; ^2^ Instituto de Ciencias Ambientales y EvolutivasFacultad de CienciasUniversidad Austral de ChileCampus Isla TejaValdiviaChile; ^3^ Instituto de Ciencias Marinas y LimnológicasFacultad de CienciasUniversidad Austral de ChileCampus Isla TejaValdiviaChile; ^4^ South American Research Group on Coastal Ecosystems (SARCE)Universidad Simón BolivarCaracasVenezuela; ^5^ Centro FONDAP de Investigación en Dinámica de Ecosistemas Marinos de Altas Latitudes (IDEAL)ValdiviaChile

**Keywords:** Community structure, consumers, disturbance, intertidal, Southern Chile, top‐down

## Abstract

The ecological consequences of human‐driven overexploitation and loss of keystone consumers are still unclear. In intertidal rocky shores over the world, the decrease of keystone macrograzers has resulted in an increase in the dominance of herbivores with smaller body (i.e., “mesograzers”), which could potentially alter community assembly and structure. Here, we experimentally tested whether mesograzers affect the structure of rocky intertidal communities during the period of early colonization after the occurrence of a disturbance. A manipulative field experiment was conducted to exclude mesograzers (i.e., juvenile chitons, small snails, amphipods, and juvenile limpets) from experimental areas in an ecosystem characterized by the overexploitation of keystone macrograzers and predators. The results of multivariate analyses suggest that mesograzers had significant effects on intertidal community structure through negative and positive effects on species abundances. Mesograzers had negative effects on filamentous algae, but positive effects on opportunistic foliose algae and barnacles. Probably, mesograzers indirectly favored the colonization of barnacles and foliose algae by removing preemptive competitors, as previously shown for other meso‐ and macrograzer species. These results strongly support the idea that small herbivores exert a firm controlling effect on the assembly process of natural communities. Therefore, changes in functional roles of top‐down controllers might have significant implications for the structure of intertidal communities.

## Introduction

The structure of natural communities can be determined by top‐down processes, in which consumers placed high in the food web modulate the patterns of relative abundance of basal species (e.g., Menge et al. 1999; O'Connor et al. 2013; He and Silliman 2015; He et al. 2015). Herbivory, as a form of consumption, plays a key role in determining the abundance, presence or absence, and distribution limits of sessile species (Lubchenco and Gaines 1981; O'Connor et al. 2011; Aguilera et al. 2015a). Evaluating these interactions has been key to understand the mechanisms behind the coexistence of species during periods of stress caused by environmental conditions or by man (Camus and Lagos 1996; Moreno 2001; O'Connor et al. 2011; Aguilera et al. 2015a; Ghedini et al. 2015).

Anthropogenic impacts on local communities have caused dramatic changes in the abundance of species and ecological interactions (e.g., Sala et al. 2000; Estes et al. 2011; Hooper et al. 2012). In particular, the pressure exerted by the overexploitation of consumers can affect the size ranges of consumers that control ecosystem dynamics (Moreno and Jaramillo 1983; Oliva and Castilla 1986; Palkovacs et al. 2012). For example, the overexploitation of large predators and herbivores (i.e., macrograzers such as fish, sea urchins, and large mollusks) on rocky shores has resulted in an increase in the abundance of smaller consumers (i.e., mesograzers such as small snails, amphipods, and juvenile limpets), likely due to competitive release (Moreno 2001; see Aguilera and Navarrete 2012a for an example of competitive release between grazers). In addition, another consequence of the functional loss of keystone predators is the increase in the abundance of competitively superior prey (Paine 1974; Durán and Castilla 1989). Forming vast patches in the intertidal zone along the rocky shores of California, South Africa, and south‐central Chile, mussels are superior competitors in these ecosystems (e.g., Paine 1974; Navarrete et al. 2005; Cole and McQuaid 2010). The mussel patches, in turn, generate a particularly complex habitat that favors the establishment and survival of smaller species such as mesograzers (e.g., Valdivia et al. 2014a). Thus, removing key consumers can cause changes to competitive interactions and habitat structure that can indirectly promote conditions necessary for the establishment of mesograzer populations.

At the population level, mesograzers can have significant effects on benthic communities. For example, a recent global meta‐analysis has shown that the experimental exclusion of mesograzers can result in a 50% increase in the abundance of primary producers (Poore et al. 2012). In intertidal rocky‐shore communities, the effect of mesograzers on sessile communities can be of a magnitude comparable to, or even greater than, the effect of macrograzers (e.g., Díaz and McQuaid 2011). In particular, Díaz and McQuaid (2011) showed that mesograzers had significant effects on the pattern of recolonization of the sessile assemblages after experimental disturbances that removed the entire experimental area. As wave‐exposed shores are often impacted by mechanical disturbances that remove biomass from the community (e.g., Menge and Sutherland 1987), mesograzers can therefore have strong effects on community structure by affecting the early stages of colonization.

Along the Chilean coast, the abundance of keystone predators and intertidal macrograzers of commercial interest (e.g., the Chilean abalone *Concholepas concholepas*, and keyhole limpets *Fissurella* spp.) has been drastically reduced (Moreno and Jaramillo 1983; Oliva and Castilla 1986; Duarte et al. 1996; Báez et al. 2004; Gelcich et al. 2010). Human‐exclusion experiments have shown that the overexploitation of keystone predators and macrograzers has caused communities originally dominated by algae to be dominated by mussels (Castilla 1999; Moreno 2001). These two community structures reflect the cascading consequences of consumers' ecological extinction; that is, their populations are driven to such small numbers that they cannot play a significant role in the community (see Estes et al. 2011 for a review at the global scale). Compelling evidence indicates significant population‐ and community‐level consequences of macrograzer loss and functional extinction in Chile and elsewhere (Moreno 2001; Martins et al. 2010; Borges et al. 2015), but further research on the potential of mesograzers to compensate this loss is still needed to achieve a mechanistic understanding of the ecological consequences of human impacts.

In this study, we experimentally test the hypothesis that, since keystone macrograzers and predators are currently overexploited and functionally extinct along the Chilean coast, mesograzers exert a firm top‐down control on the assembly and structure of the sessile benthic community during early colonization stages. To test this prediction, we used stainless steel fences to exclude mesograzers from cleared experimental areas (e.g., Díaz and McQuaid 2011). Field manipulative experiments provide an effective mean for revealing the mechanisms by which the loss or reduction of functional groups affects the dynamics of ecosystems in varying environmental conditions (e.g., Menge and Lubchenco 1981; Coleman et al. 2006; Jochum et al. 2012; O'Connor and Donohue 2013; Aguilera et al. 2015a; O'Connor et al. 2015).

## Methods

### Study site

The study was conducted in a wave‐exposed shore in southern Chile (“Calfuco”, ca. 40°S). This site is representative of fully marine, wave‐exposed southeastern Pacific shores characterized by a persistent southerly wind forcing. Long‐term (10 years) mean sea surface temperature at Calfuco ranges from 11°C in August to ca. 14°C in February. The region of the southeastern Pacific to the south of 33°S has shown during the last decade a significant cooling of coastal sea surface temperature, likely due to the intensification and migration of the Southeast Pacific Subtropical Anticyclone (Ancapichún and Garces‐Vargas 2015). The study site also conforms to open‐access shores in which no regulation on fishermen activity is enforced. Therefore, this intertidal shore can be highly impacted by anthropogenic disturbances, allowing us to have an experimental area where the effects of overfishing on the landscape are evidenced.

The experiment was conducted on high to mid‐intertidal areas, in which representative sessile organisms include opportunistic algae such as *Ulothrix* sp., *Ulva* sp., and *Pyropia orbicularis*, red corticated algae including *Mazzaella laminarioides*, chthamalid barnacles such as *Jehlius cirratus* and *Notochthamalus scabrosus*, and the purple mussel *Perumytilus purpuratus* (Jara and Moreno 1984; Moreno et al. 2001). *Perumytilus purpuratus* numerically dominates the primary substrate mainly in the mid‐intertidal zone. The macrograzers (>3 cm) in these intertidal shores include large herbivorous such as adult chitons (e.g., *Chiton granosus* and *C. magnificus*) and keyhole limpets (e.g., *Fissurella crassa*). The mesograzers (<1.5 cm), on the other hand, are abundant and include juvenile chitons, juvenile scurrinid limpets, the pulmonate gastropod *Siphonaria lessoni,* littorinids, and amphipods (Moreno 2001; Rivadeneira et al. 2002; Espoz et al. 2004). In this region, mesograzers are mainly associated with patches of *P. purpuratus*.

### Experimental design and setup

A manipulative experiment was used to test the top‐down effect of mesograzers on the structure of the benthic community. In the study site, rocky horizontal platforms (ca. 20 m long) were selected and 0.25 m^2^ areas were used as experimental units. The experimental design included three random blocks distributed along the middle intertidal. In each block, the experimental units were distributed randomly, but were limited to areas lacking large cracks and steep slopes. The temporal variation in the structure of the sessile macrobenthic assemblage in each plot was evaluated over a period of 9 months (September 2013 to May 2014). This time period was used in order to focus our analyses on the early colonization stages of these assemblages.

The design included four treatments in order to prevent or allow macro‐ and mesograzers to access the experimental plots. The treatments were (1) “grazers excluded,” in which we used stainless steel fences and copper paint to prevent all grazers to access the experimental plots; (2) “grazers present,” in which all grazers were allowed to access the plots; (3) “control,” used to detect any confounding effects (artifacts) generated by fences; and (4) “macrograzers excluded,” in which stainless steel fences were used to prevent macrograzers, but allow mesograzers, to access the plots. The grazer‐excluded treatment consisted of an open‐top fence (4.75 mm pore size), 15 cm in height and encompassing an area of 0.25 m × 0.25 m, surrounded by a 5‐cm strip of epoxy putty with copper‐based paint. The fences did not include lids in order to reduce shading effects. The grazer‐present treatment included unaltered plots marked with stainless steel screws and bolts. For the macrograzer‐excluded treatment, the same exclusion fences were used, but copper paint was not added to allow mesograzers access to the plots. Finally, the plots assigned to the control treatment were partially fenced to allow all herbivores to have access to the plot – the partial fence was also equipped with a partial strip of epoxy putty painted with copper‐based paint. A significant difference found between the control and grazer‐present treatments would serve as evidence that the fencing produced an experimental artifact (Benedetti‐Cecchi and Cinelli 1997).

Preliminary analyses showed that macrograzers were absent from the experimental area (see Fig. S1), so the macrograzer‐excluded treatment would have not contributed to detect any effect of this group on community structure. In addition, the macrograzer‐excluded treatment seemed to enhance the abundance of mesograzers, likely due to shelter provision (mean density for grazer‐present and macrograzer‐excluded plots: *Siphonaria lessoni* 33 and 92 ind. m^−2^; juvenile *Scurria* spp. 13 and 26 ind. m^−2^; and juvenile *Chiton granosus* 5 and 13 ind. m^−2^). According to these observations, we removed the macrograzer‐excluded treatment from the analysis in order to prevent artifact effects of enhanced mesograzer densities and to stream our work to the potential effects of mesograzers on community structure. Therefore, hereafter we will refer to an experimental design that encompassed three treatments: (1) grazers excluded, (2) grazers present, and (3) control.

Each of the three blocks consisted of two replicates per treatment for a total of 18 experimental units. In September 2013, all of the plots were scraped and cleaned with chisels before installing the experimental units. As purple mussel *P. purpuratus* dominated the study area (i.e., percentage cover >80%), the surface of the rock was almost free of crustose algae. The relative abundance, estimated as the percentage of total cover, of sessile species (mostly macroalgae) was estimated on a monthly basis in each experimental unit.

### Sampling method

In each plot, the percentage cover of each macrobenthic species was estimated in situ (1% accuracy) using 0.25 m^2^ quadrats that were subdivided into 25 equal fields. For each species in each field, a score of 0, 1, 2, 3, or 4 was assigned according to that species' abundance (from absence to totally covering the field; Dethier et al. 1993). The sum of the scores across 25 fields provided a composite value of abundance for each species. For mobile invertebrates, the number of individuals of each species was recorded for each quadrat.

### Statistical analysis

For each experimental unit and sessile species, we estimated the area under the curve (AUC) from a time (*x*‐axis) versus percentage cover (*y*‐axis) plot. In this way, we integrated each dependent variable over time, avoiding problems of autocorrelation in the repeated measures (e.g., Underwood 1997). AUC calculations were used as univariate measure of temporal variation of species abundances and took into account the speed and time path of colonization of sessile organisms (Svensson et al. 2005; Mrowicki et al. 2014). For example, and since all experimental plots were scraped clean at the onset of the experiment, larger AUCs indicated faster recolonization patterns over time (Mrowicki et al. 2014).

A permutations‐based multivariate mixed model (PERMANOVA; Anderson 2005) was used to test the separate and interactive effects of the grazer‐exclusion treatments (fixed factor with three levels: grazer‐excluded, present, or control) and randomized blocks (random factor with three levels) on the structure of the sessile benthic assemblage. Multivariate spatiotemporal patterns in species abundances were illustrated using a principal coordinates analysis (PCoA) that was based on Bray–Curtis similarities calculated from the AUC data. The *P*‐value of the pseudo‐*F* statistic was estimated using 10,000 permutations. After the general analysis, pairwise comparisons were performed in order to determine the effect of grazer exclusion on community structure and also to detect potential confounding effects of fences. Due to the relatively low number of possible permutations for each pairwise comparison, Monte Carlo methods were used to estimate the statistical significance. The PERMANOVA and PCoA analyses were conducted using PERMANOVA+ for the PRIMER statistical package (v6.1.14; PRIMER‐E Ltd., Plymouth, UK).

## Results

The most abundant mesograzer species found in the experimental plots were *Siphonaria lessoni*, juveniles of *Scurria* spp.*,* and juveniles of *Chiton granosus* (Fig. S1). These mesograzers were observed almost exclusively in the grazer‐present and control treatments, which corroborates the efficacy of our experimental procedure (Fig. S1). In addition, no other grazer species were observed during the experiment.

**Figure 1:**
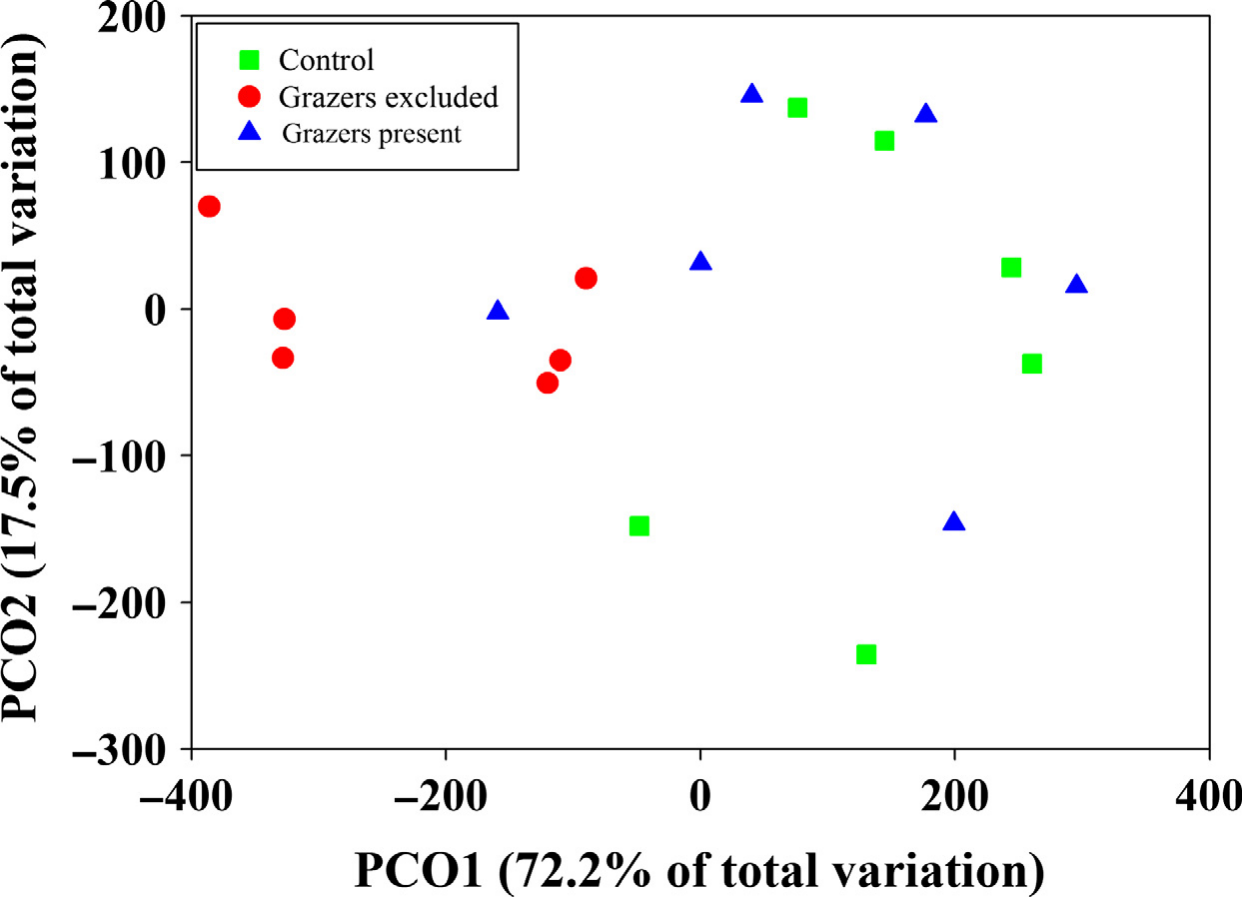
Principal coordinates analysis (PCoA) ordination showing the pattern of variation in sessile community structure as a function of grazer treatments. The PCoA ordination was based on Bray–Curtis similarities calculated from species percentage cover data integrated over time as the area under the curve.

The PCoA multivariate ordination, based on the AUCs of species abundances throughout time, showed that the grazer‐excluded plots were segregated in the multivariate space from the other two treatments, especially in the first and more important axis of variation (Fig. 1). In addition, the grazer‐present and control treatments showed a high overlap in the ordination, suggesting that confounding effects of fences were negligible (Fig. 1). When each sampling time was analyzed independently and with an alternative multivariate procedure, the pattern of segregation of the grazer‐excluded treatment still remained (Fig. S2). Accordingly, the results from the PERMANOVA supported these observations and the hypothesis that mesograzers affect community structure (Table 1); the effect of the grazer‐excluded treatment on community structure was statistically significant and provided the largest contribution to the total variation in community structure (Table 1a). We also detected a statistically significant variation among blocks, but this variation had a comparatively low contribution to the overall variation (Table 1a). The effects of grazer exclusion and blocks on community structure were independent of each other, as no statistically significant interaction between both factors was detected (Table 1a).

**Table 1:** PERMANOVA to test the effect of grazer‐exclusion treatment and randomized blocks on sessile community structure (a). Results of the post hoc statistical analysis used to test the differences between grazer‐exclusion treatments (b): grazers excluded, grazers present, and control. The statistically significant (*α *= 0.05) effects are highlighted in bold. The percentage component of variation (CV%) for each source of variation is given in (a). Due to low number unique permutations, asymptotic Monte Carlo *P*‐values were computed in (b)

(a)						
Source of variation	SS	df	MS	pseudo‐*F*	*P*_perm_	CV%
Treatment	485840	2	242920	12.68	**0.036**	46.36
Block	197350	2	98676	3.08	**0.027**	13.81
Interaction	76608	4	19152	0.60	0.829	<0.01
Residual	288390	9	32043			39.83

(b)		
Comparison	*t*	*P*_MC_
Grazers excluded, Grazers present	3.88	**0.012**
Grazers excluded, Control	7.32	**0.001**
Grazers present, Control	0.80	0.594

The comparison between grazer‐excluded and grazer‐present treatments was statistically significant, indicating an effect of the mesograzers on community structure – the statistically significant difference between the grazer‐excluded and control groups further supported this result (Table 1b). Finally, the comparison of the grazer‐present and control treatment was statistically nonsignificant, corroborating the small chances of confounding effects of fences in our experiment.

**Figure 2:**
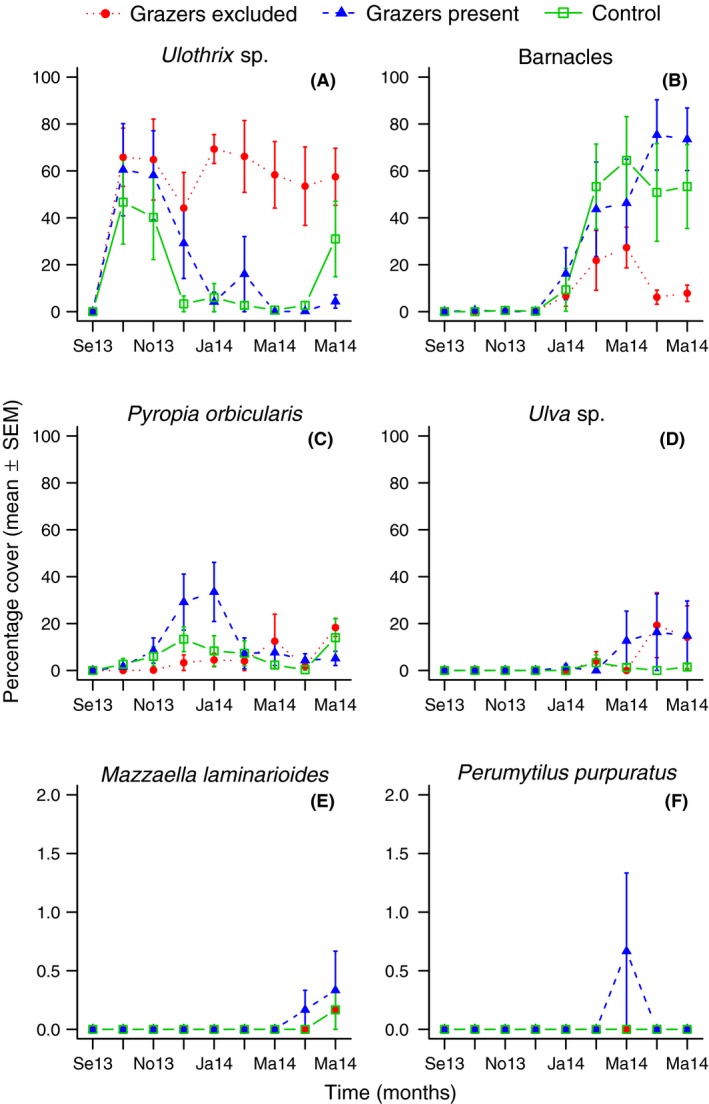
Percentage cover of sessile organisms in the grazer‐excluded, grazer‐present, and control experimental treatments during a period of 9 months of observation. Percentage covers are given as mean ± standard error of the mean (SEM). Note different scale of the *y*‐axis for *Mazzaella laminarioides* and *Perumytilus purpuratus* due to comparatively low abundances.

Two sessile taxa were the major responsible for the multivariate patterns described above. In particular, the opportunistic filamentous alga *Ulothrix* sp. and barnacles showed contrasting patterns of colonization (Fig. 2A and B). *Ulothrix* sp. was the first species to colonize the available space, but from the fourth until the ninth months it showed low abundances in the grazer‐present and control treatments (Fig. 2A). On the other hand, by the fifth month, chthamalid barnacles had abundances of 40–80% and remained with high abundances throughout the rest of the experiment in the grazer‐present and control groups (Fig. 2C).

The opportunistic red alga *Pyropia orbicularis* was found in all of the treatments, but this species showed a recruitment peak between November 2013 and February 2014 in the grazer‐present treatment and the controls in lesser extent (Fig. 2C). *Ulva* sp. and late successional species such as *Mazzaella laminarioides* and *P. purpuratus* were present in the final months of the experiment (Fig. 2D–F); *M. laminarioides* and *P. purpuratus* occurred with very low percentage covers (Fig. 2E and F).

## Discussion

Our results support the hypothesis that mesograzers significantly affect the structure of the sessile benthic assemblage. Additionally, the significant effect of blocks suggests that environmental pressures that vary locally, such as wave exposure, were also important in this experiment. Mesograzers had a strong controlling effect on the abundance of the opportunistic filamentous alga *Ulothrix* sp., but at the same time, they seemed to have positive effects on the percentage cover of chthamalid barnacles and the foliose red alga *Pyropia orbicularis*. Mesograzers therefore might affect the development of these benthic assemblages through direct and likely indirect effects on taxa competing for primary substrate. Considering that top consumers are currently being subjected to strong anthropogenic pressure, the top‐down controls on community structure in this wave‐exposed rocky‐shore system are likely exerted by small mesograzers.

Our results showed negative and positive effects of mesograzers on sessile species. The negative consumptive effect of mesograzers on *Ulothrix* sp. could have indirectly facilitated the settlement and recruitment of barnacles through reducing the strength of preemptive competition. Opportunistic and thin algae have rapid nutrient uptake rates that might allow them to monopolize the available substrate and outcompete other sessile species when disturbances are absent (e.g., Worm and Sommer 2000). A high abundance of these algae, in turn, can attract large numbers of mesograzers (Worm and Sommer 2000), which may become a negative feedback of algal population control. Positive effects of mesograzers on benthic organisms have been also reported in other systems. For example, Jaschinski and Sommer (2010) show that isopods and small gastropod can increase the productivity and growth of epiphytes in an eelgrass system. In those experiments, the isopods likely favored primary productivity by consuming the vegetal overstory and thus reducing competition for resources (Jaschinski and Sommer 2010). Similarly, isopods and amphipods can have positive effects on macroalgal growth through indirectly controlling the development of fouling epibionts (Mancinelli and Rossi 2001). Finally, mesograzers have been shown to increase substrate heterogeneity, thereby facilitating the arrival of algae that in turn increases surface for settlement (Norton and Fetter 1981; Farrell 1991). Therefore, the role of mesograzers as a structuring force in natural communities encompasses both negative (e.g., Davenport and Anderson 2007; Lewis and Anderson 2012; Poore et al. 2012) and positive effects on resources.

The dominance of mesograzers in the study region (Moreno 2001) may be due to the reduction in the abundance of competitively dominant macrograzers. Alternatively, the loss of another important component of natural communities, keystone predators, could also influence the mesograzer dominance pattern observed in this study. In agreement with classic exclusion experiments conducted elsewhere (e.g., Paine 1974), the loss of keystone predators – such as the anthropogenically impacted Chilean abalone *Cocholepas concholepas* (Gelcich et al. 2010) – results in the increase and dominance of competitively strong prey species (Moreno 2001). In several rocky‐shore communities, mussels are the top competitors and also provide a structurally complex substrate (e.g., Guiñez and Castilla 1999). An increase in surface complexity leads to an increased availability of space (Kostylev et al. 2005) biased toward small organisms (Morse et al. 1985). This, in turn, modifies both the abiotic and biotic environment and affects the community structure and ecosystem functioning (Largaespada et al. 2012). Thus, we can suggest that the overexploitation of macrograzers and keystone predators has contributed to the increase in the abundance of mesograzers.

Would these enhanced abundances of mesograzer compensate for the loss of macrograzers and keystone predators in human‐impacted communities? Manipulative studies have shown that mesograzers exert limited per capita and population effects on benthic succession when compared with those of macrograzers such as keyhole limpets and chitons (Aguilera and Navarrete 2012b). Compensation between meso‐ and macrograzers seems to be therefore unlikely in light of this evidence. Surely, the potential for compensation of small grazers will strongly depend on their population numbers, so that higher probability of compensation would be expected at higher mesograzers densities. If habitat complexity provided by dominant mussel beds favors recruitment of mesograzers, it can be expected that the high abundances of these consumers should be temporally persistent at a landscape level (see previous paragraph). As discussed above, moreover, mesograzers can have both negative and positive effects on local resources, as it has been demonstrated for macrograzers (e.g., Aguilera et al. 2015b). Provided large enough densities, thus, mesograzers would be able to compensate for the functional role of functionally lost macrograzers. In order to test this hypothesis, field experiments could be designed to compare meso‐ and macrograzer effects as functions of observed densities in human‐impacted areas (e.g., Borges et al. 2015, 2016) and the abundance of habitat‐forming species.

In our study, the significant effect of random blocks on community structure suggests that spatial processes operating at local scales of tens of meters, for example, wave exposure and shelter availability, might be relevant in this system as shown elsewhere (e.g., Denley and Underwood 1979; Fraschetti et al. 2005; Valdivia et al. 2014b). The fact that the spatial variation in community structure is usually greater at fine scales of centimeters to few meters (Fraschetti et al. 2005; Smale 2010; Valdivia et al. 2014b), hints for a pervasive patchy distribution of available shelter and environmental conditions in intertidal areas (Denny et al. 2004). However, our results also showed that the effects of mesograzers on community structure were statistically independent from this spatial variation. Although the lack of statistically significant results does not imply necessarily a lack of ecological effect (e.g., Beninger et al. 2012), we might speculate that the large densities of mesograzers observed in this system would have overridden any effect of microsite conditions on the overall grazing effect. If mesograzers can collectively have significant effects on the community structure, then future manipulative experiments should measure the effect of environmental architectural variability on the strength of herbivore interactions.

In summary, our results suggest that both negative and positive effects on sessile species compose the significant effects of mesograzers on community development and structure. The direct consumptive effect of mesograzers on pioneer species could have resulted in indirect positive effects on later colonizers through competitive release. Indirect and nontrophic interactions are pervasive in natural communities and deserve further attention (e.g., Kéfi et al. 2015). The functional extinction of macrograzers in habitats impacted by human activities could be compensated for by mesograzers if the latter reach sufficient population numbers. In addition to the context‐dependent consequences of anthropogenic disturbance described in several systems (e.g., Boyer et al. 2009; O'Connor and Donohue 2013), we suggest that enhanced abundances of mesograzers as a result of human‐driven overexploitation can have profound effects on the structure of natural communities.

## Conflict of Interest

None declared.

## Supporting information


**Figure S1.** Density of mesograzers in the grazers excluded, present, and control experimental treatments during a period of nine months of observation (from September 2013 to May 2014).
**Figure S2.** Separate non‐metric multidimensional (NMDS) ordination plots of sessile communities under grazer‐exclusion experimental treatments and sampled every month between September 2013 and May 2014.
