# Supplementary material for: Positive and negative effects of mesograzers on early‐colonizing species in an intertidal rocky‐shore community
Source: Ecol Evol. 2016 Jul 22;6(16):5761–70. doi: 10.1002/ece3.2323 (PMC4983589; doi:10.1002/ece3.2323)
Supplement: Supplementary file 1 — Figure S1. Density of mesograzers in the grazers excluded, present, and control experimental treatments during a period of nine months of observation (from September 2013 to May 2014). Figure S2. Separate non‐metric multidimensional (NMDS) ordination plots of sessile communities under grazer‐exclusion experimental treatments and sampled every month between September 2013 and May 2014. [file ECE3-6-5761-s001.docx]

Supplementary material for the manuscript entitled

**Positive and negative effects of mesograzers on early-colonizing species in an intertidal rocky-shore community**

Running head: Community-level consequences of consumer loss

Daniela Tejada-Martinez^1, 2^, Daniela N. López^1, 2^, César C. Campos^3^, Roger D. Sepúlveda^2, 4^, Nelson Valdivia ^3, 5*^

^1^Doctorado en Ciencias, mención en Ecología y Evolución, Facultad de Ciencias, Universidad Austral de Chile, Campus Isla Teja, Valdivia, Chile.

^2^Instituto de Ciencias Ambientales y Evolutivas, Facultad de Ciencias, Universidad Austral de Chile, Campus Isla Teja, Valdivia, Chile.

^3^Instituto de Ciencias Marinas y Limnológicas, Facultad de Ciencias, Universidad Austral de Chile, Campus Isla Teja, Valdivia, Chile.

^4^South American Research Group on Coastal Ecosystems (SARCE).

^5^Centro FONDAP de Investigación en Dinámica de Ecosistemas Marinos de Altas Latitudes (IDEAL)

*Corresponding author

Email: nelson.valdivia@uach.cl, Tel.: +56632221557

Fig. S1 Density of mesograzers in the experimental treatments: grazers excluded, grazer present, and control during a period of nine months of observation (from September 2013 to May 2014). Values are given as mean ± the standard error of mean (SEM). Note different scale of the y-axis across panels. The almost-nil densities in the grazer-excluded treatment throughout the experiment confirm the efficacy of the experimental procedure.

Fig. S2 Separate non-metric multidimensional (NMDS) ordination plots of sessile communities under grazer-exclusion experimental treatments and sampled every month between September 2013 and May 2014. The first sampling time (i.e. sampling 0) was omitted due to variance zero as a result of the experimental denudation of the plots. NMDS were computed and plotted with the vegan library in R environment version 3.2.4 (R Core Team 2016). The ordinations show how the grazer-excluded treatment (red circles) appears discriminated in the multivariate spaces over the experimental period.

**References**

R Core Team (2016) R: a language and environment for statistical computing. R Foundation for Statistical Computing, Vienna
